# Physical exercise and health-related quality of life in mid- to late-adulthood: a multi-group chain-mediation analysis

**DOI:** 10.3389/fpsyg.2026.1719139

**Published:** 2026-01-27

**Authors:** Qianyuan Li, Kun Wang, Zhaorui Chen

**Affiliations:** 1 School of Physical Education and Sports, Soochow University, Suzhou, China; 2 School of Foreign Languages, Central China Normal University, Wuhan, China

**Keywords:** HRQOL, mid- to late-adulthood, physical exercise, psychological resilience, self-esteem

## Abstract

**Background:**

As longevity rises in China, supporting healthy ageing has become imperative. However, the psychological pathways linking physical exercise to health-related quality of life (HRQOL), and whether they differ by gender, remain insufficiently understood.

**Methods:**

Using data from 762 adults aged 45 + in Shandong and Hunan, we examined how physical exercise relates to HRQOL and whether psychological resources explain this link. A cross-sectional survey was analyzed with confirmatory factor analysis and structural equation modeling. Bias-corrected bootstraps were used to test indirect and sequential pathways, and multiple-group models compared men and women.

**Results:**

Physical exercise showed a positive direct association with HRQOL. This association was jointly mediated by self-esteem and psychological resilience, with the mediators operating both independently and sequentially. The overall pattern was broadly comparable across genders; however, the direct association was stronger among men (*β* = 0.315) than among women (*β* = 0.115).

**Conclusion:**

The findings clarify cognitive–affective mechanisms connecting activity to later-life wellbeing and indicate that combining regular exercise with programs that foster self-esteem and psychological resilience is associated with health-related quality of life among middle-aged and older adults. Combining opportunities for regular exercise with programs that cultivate self-esteem and resilience could be an efficient avenue for enhancing HRQOL among middle-aged and older adults.

## 1 Introduction

The current life expectancy in China has reached 78.6 years, more than double the 35 years at the founding of the People’s Republic, and is expected to surpass the 80-year threshold within the next decade (Bai et al., 2023). Unlike younger groups, middle-aged and older Chinese adults face poor health prospects (Li et al., 2025a); over half live with chronic diseases and multimorbidity, leading to crises that reduce their healthy and subjective life expectancy (Tang et al., 2024). The concept of healthy ageing goes beyond biological longevity, placing stronger emphasis on improving life quality by maximizing “healthy life years” and maintaining well-being throughout later adulthood (Liu et al., 2025; Offerman et al., 2023).

HRQOL, a key facet of overall quality of life, reflects individuals’ multidimensional self-appraisals of their health status. It is commonly assessed with self-report tools to gauge health and life quality among mid- and later-life populations (Karimi and Brazier, 2016). Recognized as a cornerstone of successful ageing (Hajek and König, 2024), HRQOL generally declines with advancing age (Hwang et al., 2023) and fell further during COVID-19 as many older adults faced intensified social isolation and loneliness (Sayin Kasar and Karaman, 2021). Improving HRQOL can both reduce pressures on health and social-care systems and enhance well-being and life satisfaction (Santhalingam et al., 2022; Singh and Dixit, 2010).

Relative to genetic risk profiles, lifestyle choices account for a larger share of variation in longevity (Li et al., 2025b). Evidence from the Chinese Longitudinal Healthy Longevity Survey also shows that, among behaviors associated with better HRQOL, being physically active is particularly salient (Chen et al., 2024). Studies of middle- and later-life adults, including those at risk of disability, find that the activity–HRQOL association often exceeds the effects of sociodemographic characteristics and chronic conditions, and that movement-focused interventions yield meaningful health gains in this population (Groessl et al., 2007). This association holds across measurement modes, whether self-report or device-based, such that higher activity corresponds to better HRQOL (Anokye et al., 2012).

This study is theoretically grounded in the cognitive-affective-conative framework, an integrative theory in psychology used to systematically explain the relationship between human behavior and psychological processes (Hilgard, 1980). Its core proposition is that psychological functioning consists of cognition, affect (emotional experience), and conation, which dynamically interact to jointly influence behavior (Mayer et al., 1997). Applied to this study, the theory explains how physical exercise influences HRQOL indirectly through self-esteem and psychological resilience. At the cognitive level, engaging in exercise fosters healthy habits and positive self-perceptions, improving functional ability and daily self-care, which in turn strengthens self-esteem. At the affective level, higher self-esteem generates positive experiences such as achievement and belonging, enhancing emotions and building resilience. At the conative level, with greater self-esteem and resilience, middle-aged and older adults are better equipped to manage age-related chronic conditions and cope with unexpected challenges, thereby improving their self-rated HRQOL.

Unlike earlier research that focused on direct physiological pathways linking exercise and HRQOL, such as declines in HRQOL among inactive sarcopenic patients (Sun et al., 2019) or improvements from resistance training that increases muscle mass and strength (Haraldstad et al., 2017), this study emphasizes psychological mechanisms. This emphasis is warranted because, among Chinese older adults, HRQOL is shaped not only by chronic illness but also by lifestyle and cognitive factors (Chen et al., 2020), and large-scale surveys suggest that psychological variables make the greatest contribution to HRQOL outcomes (Gaspar et al., 2012). Although several studies have examined psychological factors mediating the relationship between physical exercise and HRQOL in Chinese populations, these studies generally lack a well-developed theoretical framework (Li Q. et al., 2024; Li X. et al., 2024). Drawing on the cognitive–affective–conative framework, the present study systematically examines the mediating roles of self-esteem and psychological resilience in the association between physical exercise and HRQOL among middle-aged and older adults.

Moreover, starting exercise in midlife leads to higher HRQOL, with benefits even when begun at age 55 (Nguyen et al., 2024). Cognitive correlates change across the life course: some (e.g., subjective well-being) follow a U-shaped pattern, whereas others (such as self-esteem) show an inverted-U, highlighting the salience of midlife (Blanchflower and Oswald, 2008; Kusumastuti et al., 2016). Evidence also indicates sex-based heterogeneity in how these factors relate to HRQOL (Hajek et al., 2016). Building on this and to extend the application of the cognitive–affective–conative framework to the domain of physical exercise among middle-aged and older adults, our objectives are to: (i) quantify links among physical activity, self-esteem, psychological resilience and HRQOL in adults from midlife onwards; (ii) within the cognitive–affective–conative framework, evaluate whether self-esteem and resilience transmit the effects of activity to HRQOL—both as sequential and as parallel mediators; and (iii) test cross-sex applicability of the model and compare path strengths for men and women.

We organize the article into six parts—beginning with the research background and related literature, then setting out our hypotheses and methods, followed by the analysis and results, and ending with the conclusions and contributions.

## 2 Literature review

### 2.1 Concepts

#### 2.1.1 Physical exercise

Exercise is typically goal-directed—it is planned, organized and repetitive, with an explicit aim of improving fitness and supporting psychological well-being (Caspersen et al., 1985; Teixeira et al., 2012). Drawing on Dasso (2019), exercise denotes the structured and purposeful portion of those movements designed to build or maintain physical fitness. For the purposes of this study, we define exercise as voluntary leisure-time participation by middle-aged and older adults with specified intensity, frequency and duration, undertaken to promote both physical and mental health.

#### 2.1.2 Self-esteem

Rosenberg (1965) characterized self-esteem as people’s favorable or unfavorable evaluations of themselves. Informed by social identity theory, Du et al. (2017) proposed a three-part structure comprising personal, relational and collective self-esteem, illustrating cultural variation in how self-esteem is organized. Aligned with this perspective, the present study concentrates on personal self-esteem, understood as middle-aged and older adults’ self-image, including feelings of appreciation, respect, recognition and confidence in their own abilities.

#### 2.1.3 Psychological resilience

Psychological resilience is a universal potential inherent in individuals, reflecting their capacity to adapt when confronted with adversity, trauma, or significant stress (Masten, 2007). Moreover, resilience demonstrates strong contextual specificity and temporal dynamics, with its mechanisms varying across developmental stages and environmental changes (Luthar et al., 2000). In summary, in this study, psychological resilience is identified as the effective adaptive and coping capacity displayed by middle-aged and older adults when facing stress or adversity, namely, adaptability in the face of difficulties.

#### 2.1.4 HRQOL

HRQOL is a health-specific subset of overall quality of life and was originally conceptualized with an emphasis on physiological indicators, such as morbidity and physical functional capacity (Torrance, 1987). In practice, however, physical, psychological, and social domains are interrelated, and this interactive structure indicates that focusing solely on single physiological indicators is insufficient; subjective evaluations at the psychological and social levels are equally important (Hart and Buck, 2019; Rony et al., 2024). Accordingly, in this study we conceptualize HRQOL among middle-aged and older adults as a multidimensional, self-reported appraisal of health that integrates physical, mental and social functioning and reflects overall satisfaction with these facets.

### 2.2 Hypotheses development

For midlife women, exercise can ease menopausal complaints while strengthening adaptive psychological dispositions, including self-esteem (Dąbrowska-Galas and Dąbrowska, 2021). More generally, sustained participation in physical activity, particularly through longer-term programs, is linked to marked gains in self-esteem in later-life populations (Park et al., 2014). Elevated self-esteem, often accompanied by higher self-efficacy, functions as a resource that protects HRQOL by buffering stress (Mikkelsen et al., 2020). Findings from Chinese samples likewise show that regular activity enhances subjective well-being via gains in self-esteem across both youth and older adults (R. Chen et al., 2022; Shang et al., 2021). Given that subjective well-being is a core facet of, and conceptually overlaps with, HRQOL (Camfield and Skevington, 2008), self-esteem represents a key pathway through which exercise is associated with HRQOL. Recent evidence further indicates that moderate-intensity exercise can indirectly bolster HRQOL and later-life mental health through improvements in self-esteem (Mijalković et al., 2025).

Low resilience frequently co-occurs with mental health problems, most notably depression, which undermine HRQOL (van Kessel et al., 2014). Although chronic illness and functional loss are prevalent in old age, resilience often persists. Physical activity appears to bolster this resilience, and the gains scale with the degree of engagement (Toth et al., 2023). Even in frail older adults, participation in exercise interventions has been found to improve psychological flexibility (Rossi et al., 2021). Indeed, by satisfying basic psychological needs and promoting adaptive self-regulatory patterns, physical exercise can improve individuals’ levels of self-esteem and resilience (Diotaiuti et al., 2021). Research shows that resilience mediates the link between exercise and psychological benefits. Physical activity raises life satisfaction both directly and through enhanced resilience (Deng et al., 2023). Sustained participation also cultivates perceptions of perseverance and self-care, which in turn strengthen psychological wellness in later life (Wermelinger Ávila et al., 2022). Notably, both life satisfaction and mental health are integral subsets of HRQOL.

Framed by a cognitive–affective–conative perspective, we contend that exercise improves physical functioning in mid- and later-life adults, thereby elevating self-esteem; higher self-esteem supplies the psychological capital needed to cultivate resilience; and greater resilience equips older adults to manage both current and unforeseen age-related risks, indirectly enhancing HRQOL. Earlier work often modeled resilience and self-esteem as independent, parallel mediators between antecedents and outcomes (Lin et al., 2024). In reality the constructs are tightly coupled: they correlate strongly, and self-esteem frequently precedes and predicts resilience. Experiences that lift self-esteem can therefore also raise resilience (Kocatürk and Çiçek, 2023). Empirical evidence shows that resilience carries the effect of self-efficacy to mental health (Qin et al., 2023), and self-efficacy is closely aligned with self-esteem (Lane et al., 2004). Against this backdrop, physical activity can increase vitality, self-esteem and life satisfaction, which in turn dampens negative affect, enhances positive emotions and improves adaptation (Rodrigues et al., 2022).

Previous studies have suggested that gender-specific interventions may be necessary to improve HRQOL, as both self-esteem and HRQOL differ significantly between men and women (Mikkelsen et al., 2020). Although resilience exhibits contextual specificity and temporal dynamics, and women generally report higher levels of resilience than men, physical exercise exerts a significant effect on resilience regardless of gender, age, or education level (Luthar et al., 2000; Masley et al., 2009; Mendes et al., 2023). The role of gender in links among exercise, self-esteem, resilience and HRQOL remains unsettled. Drawing on prior work, we posit the following mediation hypotheses:

Based on this literature, the study proposes:

*H1*: Engagement in physical exercise is positively associated with HRQOL among adults in mid- and later life.

*H2*: Self-esteem transmits the effect of physical exercise on HRQOL among adults in mid- and later life.

*H3*: Psychological resilience mediates the association between physical exercise and HRQOL among adults from midlife onwards.

*H4a*: Self-esteem and psychological resilience act jointly to transmit the effect of physical exercise to HRQOL in adults from midlife onwards.

*H4b*: Among men in mid- and later life, self-esteem and psychological resilience jointly mediate the association between physical exercise and HRQOL.

*H4c*: Among women in mid- and later life, self-esteem and psychological resilience jointly mediate the association between physical exercise and HRQOL.

**Figure 1:**
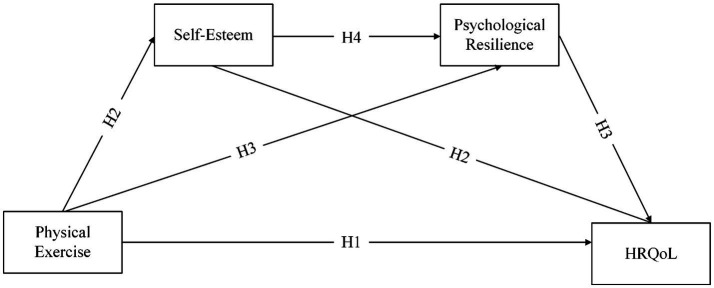
Theoretical model.

Figure 1 depicts the hypothesized pathways.

## 3 Methods

### 3.1 Participants and procedures

This study focused on middle-aged and older adults (≥45 years) with at least one month of exercise experience, as active ageing preparation should ideally begin in midlife. Following WHO classification: 45–59 years = middle-aged; 60–74 years = older adults; ≥75 years = oldest-old. Shandong and Hunan Provinces were chosen as study sites due to their large elderly populations, both exceeding the national average (Shandong: 21.22 million, 20.9%; Hunan: 14.60 million, 13.82%).

Data were collected between July and September 2024 using snowball and convenience sampling. To accommodate limited use of smart devices, we administered the survey in both digital and paper formats. Participants were briefed on the study purpose and informed participation was voluntary. On completion, respondents received either mobile top-up credit or a gym pass. Of 810 questionnaires distributed, 782 were returned; after excluding invalid responses, 762 valid samples were retained, yielding a 97.4% effective response rate.

To ensure data quality and reduce the likelihood of outliers and extreme response patterns, an initial data-cleaning procedure was conducted after questionnaire collection. Specifically, we: (1) screened and removed questionnaires with uniform responses across all items; (2) excluded questionnaires completed in an implausibly short time; and (3) included reverse-coded items to check logical consistency with corresponding positively worded items.

To determine the required sample size for structural equation modeling, we applied the formula proposed by Scheaffer et al. (1990).


n=N/(N(δ)^2+1)

Where n represents the target sample size, N denotes the known total population size (here referring to the middle-aged and older population in China), and δ represents the acceptable sampling error. Using a 95% confidence level and a 5% margin of error, the estimated minimum sample size was approximately 400. The final analytic sample of 762 respondents after data cleaning therefore exceeded the required threshold.

As shown in Table 1, the sample characteristics were as follows: (1) the gender distribution was relatively balanced, with 51.57% men and 48.43% women, approximating a 1:1 ratio; (2) nearly half of the respondents (384 individuals, 50.39%) were classified as middle-aged; and (3) the majority reported a preference for non-competitive forms of physical activity (554 individuals), such as jogging, square dancing, and Tai Chi. Percentages reported in the sample description and subsequent tables were rounded to the nearest whole number.

**Table 1:** Demographic characteristics.

Variable	Category	*n*	%
Gender	Male	393	51.57
	Female	369	48.43
Age	45–59 years	384	50.39
	60–74 years	262	34.38
	75 years and above	116	15.22
Employment status	Unemployed	227	29.79
	Self-employed	287	37.66
	Employed	248	32.55
Educational attainment	Primary school or below	136	17.85
	Junior high school	152	19.95
	Senior high school or vocational secondary school	163	21.39
	College diploma or bachelor’s degree	159	20.87
	Master’s degree	78	10.24
	Doctoral degree	74	9.71
Household registration	Rural	369	48.43
	Urban	393	51.57
Exercise items	Walking, jogging	162	21.26
	Travel, excursions	134	17.59
	Dance-based activities (e.g., square dancing)	114	14.96
	Ball games	78	10.24
	Rope skipping	74	9.71
	Traditional fitness practices (e.g., Tai Chi, Yijin Jing, broadcast gymnastics)	70	9.19
	Fitness equipment activities	52	6.82
	Swimming	46	6.04
	Others	32	4.20

### 3.2 Instruments

This study employed a questionnaire comprising five sections. The first collected demographic information (e.g., gender, age, type of exercise). The second applied the revised Physical Exercise Scale (Liang, 1994), including three items (Cronbach’s *α* = 0.79; KMO = 0.704). The third used Rosenberg (1965) Self-Esteem Scale (SES), with 10 items—five positively scored and five reverse-coded (Cronbach’s α = 0.931; KMO = 0.960). The fourth employed the 25-item Connor–Davidson Resilience Scale (CD-RISC) (Connor and Davidson, 2003), covering strength, optimism, and tenacity (Cronbach’s α = 0.918; KMO = 0.940). The fifth section assessed HRQOL using the EQ-5D-5L Scale (Herdman et al., 2011), comprising five items scored via the Chinese value set (Luo et al., 2017). In this valuation system, the constant term is set to 1, and the response levels for each dimension are treated as additive decrements. Table 2 reports the corresponding coefficient values derived from Chinese population norms. Utility values were calculated as:


U=1−(MOn+SCn+UAn+PDn+ADn),n=1,2,3,4,5

Ranging from −0.391 to 1.000 (Cronbach’s α = 0.89; KMO = 0.884). For example, if a respondent selected level 5 for all five EQ-5D-5L dimensions, the calculation would be:


U=1−(MO5+SC5+UA5+PD5+AD5)

U=1−(0.345+0.253+0.233+0.302+0.258)=−0.391

**Table 2:** Chinese value set for the EQ-5D-5L health utility index.

Level	MO	SC	UA	PD	AD
1	0	0	0	0	0
2	0.066	0.048	0.045	0.058	0.049
3	0.158	0.116	0.107	0.138	0.118
4	0.287	0.210	0.194	0.252	0.215
5	0.345	0.253	0.233	0.302	0.258

All scales have been validated in Chinese populations (Deng et al., 2023; Kong et al., 2012; Luo et al., 2017; Yu and Zhang, 2007).

### 3.3 Data analysis

We estimated a structural equation model (SEM) in AMOS 23 to evaluate links among physical exercise, self-esteem, psychological resilience and HRQOL in mid- to later-life adults. Using the standard two-stage procedure, we first examined the measurement model for reliability and validity, then assessed structural fit and path estimates, including indirect and sequential (chain) effects. To explore sex differences, multigroup chain-mediation models were fitted separately for men and for women.

To gauge potential common-method variance (CMV) from self-reports, we applied Harman’s single-factor procedure. Six factors were extracted, with the largest accounting for 26.34% of variance—below the customary 30% criterion (Podsakoff and Organ, 1986). We also ran a single-factor CFA; shifts in global fit were negligible (RMSEA +0.001, CMIN/df + 0.008, GFI + 0.003, NFI + 0.002, IFI + 0.001), each under the 0.10 cut-off (Richardson et al., 2009).

In addition, we used MATLAB to compute the heterotrait–monotrait (HTMT) ratios among the four constructs. As shown in Table 3, all HTMT values were below the stringent threshold of 0.85 (range: 0.286–0.569), indicating satisfactory discriminant validity among the constructs (Gold et al., 2001). Collectively, these diagnostics indicate that CMV was unlikely to pose a serious problem in this study.

**Table 3:** HTMT analysis.

Variable	PE	SE	PR	HRQOL
PE	1			
SE	0. 424	1		
PR	0.373	0.286	1	
HRQOL	0.569	0.487	0.424	1

## 4 Results

### 4.1 Measurement model

We ran a CFA in AMOS v23 to appraise the latent measures’ reliability and validity. As reported in Table 4, all scales showed strong internal consistency (Cronbach’s *α* > 0.70) and acceptable convergent validity (AVE > 0.50; CR > 0.70). Standardized factor loadings ranged from 0.707 to 0.919, and the confidence intervals for all standardized loadings were entirely above 0.50, further supporting the measurement structure. Discriminant validity was also met: for every construct, the square root of its AVE exceeded the corresponding inter-construct correlations. The R^2^ values indicate that physical exercise explained 11.7% of the variance in self-esteem; physical exercise and self-esteem jointly explained 11.1% of the variance in psychological resilience; and the full model—including physical exercise, self-esteem, and psychological resilience—accounted for 32.8% of the variance in HRQOL (see Table 5).

**Table 4:** Reliability and validity.

Items	Factor loadings	Cronbach’s α	CR	AVE	95% CI
Physical exercise (PE)		0.790	0.829	0.618	
PE1	0.805				0.723–0.882
PE2	0.753				0.668–0.830
PE3	0.799				0.715–0.875
Self-esteem (SE)		0.931	0.933	0.582	
SE1	0.919				0.856–0.961
SE2	0.722				0.621–0.808
SE3	0.756				0.662–0.836
SE4	0.707				0.610–0.791
SE5	0.726				0.627–0.812
SE6	0.736				0.639–0.820
SE7	0.742				0.646–0.825
SE8	0.751				0.657–0.832
SE9	0.785				0.695–0.860
SE10	0.764				0.672–0.842
Psychological resilience (PR)		0.918	0.769	0.626	
PRP	0.725				0.624–0.810
PRO	0.770				0.676–0.848
PRT	0.852				0.779–0.911
HRQOL		0.890	0.888	0.614	
HRQOL1	0.818				0.735–0.886
HRQOL2	0.774				0.684–0.850
HRQOL3	0.768				0.677–0.845
HRQOL4	0.747				0.653–0.827
HRQOL5	0.810				0.726–0.880

95% CI = confidence intervals for standardized loadings.

**Table 5:** Pearson correlation.

Variable	Construct	PE	SE	PR	HRQOL	R^2^
Overall sample	PE	(0.786)				
	SE	0.343 **	(0.763)			0.117
	PR	0.282 **	0.264 **	(0.791)		0.111
	HRQOL	0.431 **	0.435 **	0.374 **	(0.783)	0.324
Male group	PE	(0.790)				
	SE	0.348 **	(0.774)			
	PR	0.311 **	0.313 **	(0.756)		
	HRQOL	0.516 **	0.479 **	0.421 **	(0.732)	
Female group	PE	(0.767)				
	SE	0.306 **	(0.892)			
	PR	0.299 **	0.271 **	(0.762)		
	HRQOL	0.315 **	0.380 **	0.352 **	(0.693)	

Diagonal values are the square roots of the AVE, while off-diagonal values indicate Pearson correlations between constructs. ** *p* < 0.01.

### 4.2 Structural model

With the measurement model validated, we estimated the structural paths in AMOS v23 using 5,000 bootstrap draws. Fit was satisfactory for the full sample and for both sexes: overall χ^2^/df = 1.484, GFI = 0.996, AGFI = 0.986, NFI = 0.988, TLI = 0.990, RFI = 0.970, RMSEA = 0.025, SRMR = 0.016; male χ^2^/df = 1.035, GFI = 0.995, AGFI = 0.982, NFI = 0.987, TLI = 0.999, RFI = 0.968, RMSEA = 0.010, SRMR = 0.018; female χ^2^/df = 1.285, GFI = 0.993, AGFI = 0.976, NFI = 0.973, TLI = 0.984, RFI = 0.932, RMSEA = 0.028, SRMR = 0.024. All indices satisfied conventional criteria (χ^2^/df < 3; GFI/AGFI/NFI/CFI/TLI/RFI > 0.90; RMSEA < 0.06; SRMR< 0.05), indicating strong model fit.

**Figure 2:**
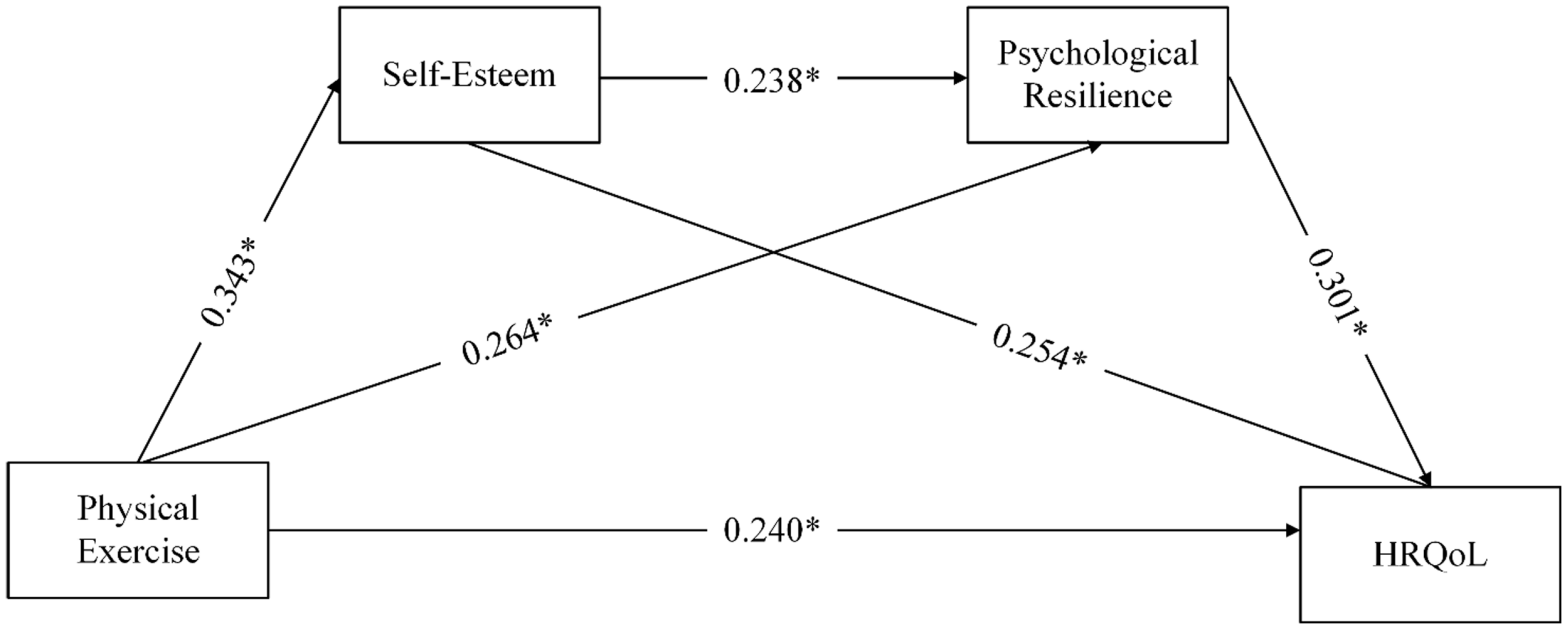
Structural path model. *p < 0.05.

Pearson correlations (Table 5) also evidenced significant links among constructs. As depicted in Figure 2, physical exercise was positively related to HRQOL (*β* = 0.240, *p* < 0.05), thereby supporting H1. This standardized coefficient indicates that a one–standard deviation increase in physical exercise was associated with a 0.24–standard deviation increase in HRQOL.

As summarized in Table 6, bootstrapped mediation tests (5,000 draws; 95% bias-corrected CIs) revealed significant indirect paths. The exercise → HRQOL link operated through self-esteem (indirect effect = 0.087, accounting for 26.61% of the total effect; SE = 0.013, 95% CI [0.063, 0.115], *p* < 0.001) and through psychological resilience (indirect effect = 0.080, accounting for 24.76% of the total effect; SE = 0.021, 95% CI [0.044, 0.127], *p* < 0.001). The sequential route via self-esteem then resilience was also significant (indirect effect = 0.025, accounting for 9.43% of the total effect; SE = 0.006, 95% CI [0.014, 0.040], *p* < 0.001). Effects were comparable by sex—men: 0.029 (SE = 0.009, 95% CI [0.014, 0.050], *p* < 0.001); women: 0.027 (SE = 0.011, 95% CI [0.010, 0.053], *p* < 0.05). These outcomes support Hypotheses 2, 3, and 4a–4c.

**Table 6:** Standardized indirect effect.

Path	Point estimate	Product of coefficients		Bootstrapping		
				Bias-corrected 95% CI		Two-tailed significance
		SE	Z	Lower	Upper	
PE → SE → HRQOL	0.087	0.013	6.692	0.063	0.115	*p* < 0.001
PE → PR → HRQOL	0.080	0.021	3.810	0.044	0.127	*p* < 0.001
PE → SE → PR → HRQOL	0.025	0.006	4.167	0.014	0.040	*p* < 0.001
Male group: PE → SE → PR → HRQOL	0.029	0.009	3.222	0.014	0.050	*p* < 0.001
Female group: PE → SE → PR → HRQOL	0.027	0.011	2.455	0.010	0.053	*p* < 0.05

### 4.3 Comparison of model path differences

After establishing invariance of the structural model across sexes, we compared path coefficients. To examine sex differences, we conducted multi-group structural equation modeling, estimating the model separately for male and female samples. Group differences were assessed using the critical ratio (CR), calculated as the difference between the two path coefficients divided by their pooled standard error. As reported in Table 7, only the direct path from physical exercise to HRQOL differed significantly by sex (CR = −1.979, |CR| > 1.96). This effect was stronger for men (*β* = 0.315) than for women (*β* = 0.115), with a difference of Δβ = 0.200, indicating sex-based moderation of this relationship. Differences in the remaining paths were observed but did not reach statistical significance.

**Table 7:** Gender differences in path coefficients of the structural model.

Path	Gender	Path coefficient	SE	CR	Δβ (Male–Female)	Critical ratio
PE → SE	Male	0.348*	0.012	7.341	0.042	0.599
	Female	0.306*	0.016	6,174		
SE → PR	Male	0.283*	0.041	4.373	0.020	−1.148
	Female	0.263*	0.033	3.684		
PE → PR	Male	0.277*	0.010	4.290	−0.028	0.032
	Female	0.305*	0.011	4.021		
PE → HRQOL	Male	0.315*	0.001	7.018	0.200	−1.979*
	Female	0.115*	0.001	2.124		
SE → HRQOL	Male	0.257*	0.002	5.716	0.034	−0.481
	Female	0.223*	0.002	4.200		
PR → HRQOL	Male	0.297*	0.004	4.847	−0.044	1.385
	Female	0.341*	0.007	4.096		

* *p* < 0.05.

## 5 Discussion

Using survey data from Shandong and Hunan, we examined links between physical activity, self-esteem, psychological resilience and HRQOL among adults from midlife onwards. Extending recent work in Chinese samples (Chen et al., 2024; Li Q. et al., 2024) and adopting a cognitive–affective–conative lens, our analyses showed that self-esteem and resilience both mediate the pathway from exercise to HRQOL. These findings not only enhance understanding of how exercise is associated with HRQOL in later life but also extend the application of the cognitive–affective–conative framework. They further offer several directions for discussion.

Although physical exercise explains only a modest proportion of the variance in HRQOL, both prior research and the present findings confirm its statistically significant and substantively meaningful association with HRQOL among older adults (Jurakić et al., 2010). After dividing the sample by gender, the relationship between exercise and HRQOL remained significant, thereby extending earlier studies that had focused exclusively on older men (Haraldstad et al., 2017). However, comparisons of path coefficients indicated that exercise had a stronger direct association with HRQOL among men (*β* = 0.315) than among women (*β* = 0.115).

This disparity may partly reflect physiological differences, as physical discomfort or pain may constrain women’s ability to fully engage in exercise, thereby shaping their HRQOL evaluations (Vuillemin et al., 2005). At the same time, China’s rapid social transformation places modern women under multiple, overlapping pressures related to career development, family responsibilities, and societal expectations. The combined impact of these demands may weaken the association between physical exercise and subjectively evaluated HRQOL among women (Xiao et al., 2025). Moreover, gender differences in exercise types, intensity, and voluntariness may also account for the variation in HRQOL outcomes (Jurakić et al., 2010). In the Chinese context, prevailing social norms differ in expectations regarding whether and how men and women should engage in physical activity. Traditional views tend to endorse men’s participation in exercise—particularly outdoor activities—as a means of displaying social and economic status, allowing exercise to yield greater social and emotional returns for men. In contrast, women’s participation in exercise is often constrained by gender stereotypes that emphasize gentleness over physical strength, which may help explain why exercise is more strongly associated with men’s subjectively evaluated HRQOL (Ye et al., 2025).

In addition, during the aging process, women tend to exhibit greater emotional sensitivity and more pessimistic self-appraisals of their health status, often requiring emotional support rather than the primarily physical stimulation provided by exercise (Deng et al., 2025). Furthermore, within the Chinese cultural context, older adults show a smaller gain in HRQOL from exercise compared with adolescents (Li Q. et al., 2024). This observation is consistent with Bize et al. (2007), who cautioned that activity–HRQOL relations seen in older populations may not carry over to youth.

We find that self-esteem and psychological resilience sequentially mediate the association between physical exercise and HRQOL in later life among middle-aged and older adults, indicating a chained mediating process. These patterns are consistent with earlier studies highlighting both constructs as positive determinants and mediators of the exercise–HRQOL link (Chen et al., 2022; Diotaiuti et al., 2021; Mikkelsen et al., 2020, 2021; Shang et al., 2021; Tecson et al., 2019; Wermelinger Ávila et al., 2022; Zhong et al., 2025). Moreover, grounded in the cyclical logic of the cognitive–affective–conative framework, our findings illustrate how exercise, self-esteem, resilience, and HRQOL form a reinforcing upward spiral. Conation provides the initial motivational impetus that drives engagement in physical activity. This behavior then yields positive outcomes that foster favorable cognitions (e.g., “I can do it”) and positive emotions, thereby enhancing self-esteem (Dąbrowska-Galas and Dąbrowska, 2021; Park et al., 2014). Elevated self-esteem then strengthens resilience (Kocatürk and Çiçek, 2023; Rodrigues et al., 2022). With greater resilience, older adults are more likely to adopt proactive coping strategies (conation) and healthier cognitive appraisals when confronting age-related challenges, which ultimately mediates the association between physical exercise and HRQOL (Fernandez et al., 2015; Tecson et al., 2019; Wermelinger Ávila et al., 2022).

Previous studies have placed greater emphasis on the links between women’s physical activity and cognitive factors (Dąbrowska-Galas and Dąbrowska, 2021). Women often face more adversity and challenges across the life course, including stressful events such as family caregiving and financial pressures, which may help them develop higher resilience levels (Manning, 2014). As they age, women also tend to exhibit stronger psychological adaptability and resilience than men (Netuveli et al., 2008). Indeed, gender differences are evident in both self-esteem and HRQOL (Mikkelsen et al., 2020), and prior research shows that the effects of cognitive factors on HRQOL vary between men and women (Hajek et al., 2016).

To address these differences, we re-estimated the model separately for men and women. The results demonstrated satisfactory model fit in both groups, with self-esteem and psychological resilience sequentially mediating the association between physical exercise and HRQOL among middle-aged and older adults. Path comparisons revealed no significant gender differences in either the single mediating or the chained mediating effects, suggesting that the roles of self-esteem and resilience in linking exercise to HRQOL are robust across genders. Although certain cognitive factors are shaped by gender and may differ in their relative importance for HRQOL (Hajek et al., 2016), the indirect and sequential pathways involving self-esteem and resilience appear stable for both men and women (Luthar et al., 2000; Masley et al., 2009; Mendes et al., 2023), exerting consistent positive effects.

Against the backdrop of China’s dual context of enduring traditional cultural norms and rapid social transformation—and given the stable associations among physical exercise, self-esteem, psychological resilience, and HRQOL alongside distinctive gender differences—we advocate for improving the broader sociocultural climate that generates gender-based expectations. Women should not be presupposed as primarily service-oriented family caregivers; rather, they should be equally encouraged to participate in physical exercise as a means of expressing physical capability and competitiveness, which are closely tied to their self-esteem and psychological resilience. At the same time, contemporary Chinese society continues to hold gender-differentiated conceptions of successful aging, tending to valorize physical strength in men and gentleness in women. Such norms make men more likely to view physical capacity as a central marker of HRQOL. Although women may place greater emphasis on emotional support, the association between physical exercise and their HRQOL—as well as the mediating roles of self-esteem and resilience—should not be overlooked. Efforts are therefore needed to challenge these sociocultural norms and to promote equal opportunities for physical activity participation among older adults of all genders.

## 6 Limitation

Because of data-collection limits, we used a cross-sectional design; reliance on a single measurement occasion precludes causal inference and limits conclusions regarding reverse causality. In addition, the use of convenience and snowball sampling may have introduced selection bias, thereby constraining the generalizability of the findings. The heterogeneous incentives provided (mobile phone top-up credit versus gym passes) may also have functioned as potential confounding motivational factors. Moreover, the three-item self-report measure of physical exercise is susceptible to subjective recall bias; future studies could employ accelerometers or other device-based measures to obtain more objective assessments. At the same time, although the present study focused on psychological mediating pathways, the EQ-5D-5L may underestimate respondents’ psychological domain scores. Accordingly, we focused on associations between physical exercise and HRQOL in mid- to later-life adults and examined sex differences. However, the analyses did not incorporate a broader range of sociodemographic and health-related control variables. Future research could include additional sociodemographic covariates and stratify participants by comorbidity status or severity of chronic conditions to enable more precise tests of the proposed mediating pathways.

In addition, as the data were primarily derived from self-reported questionnaires, there remains a possibility of inflated social desirability responses or common method bias, despite our use of reverse-coded items at the data collection stage and methodological testing during analysis. Finally, while a sex-based difference emerged for the direct effect of exercise on HRQOL, the mediation pathways through self-esteem and psychological resilience—whether single or chained—showed no gender variation. Future research might fruitfully consider other variables, including subjective well-being and perceived stress, that may influence this linkage. Under appropriate control of *α* inflation associated with multiple mediation testing, sensitivity analyses (e.g., adding control variables, incorporating depression and perceived stress into the model) and longitudinal designs using device-based measurements would help clarify the causal relationships among the study variables.

## 7 Conclusion

This study shows that regular exercise enables mid- and later-life adults to maintain HRQOL, with self-esteem and psychological resilience acting as the principal transmission mechanisms. Effects were comparable for men and women, underscoring exercise as a broadly applicable route to later-life wellbeing. Given the sizeable impact of activity on HRQOL, family, community and park facilities should be upgraded for age-friendliness. Policies that expand access to venues and cultivate positive, supportive settings are pivotal for sustaining participation among older people.

## Data Availability

The raw data supporting the conclusions of this article will be made available by the authors, without undue reservation.
